# Strengthening Faculty Development through Regional and Global Collaboration: An Innovative Virtual Program in Cambodia

**DOI:** 10.5334/aogh.4660

**Published:** 2025-06-10

**Authors:** Vannary Yi, Khoa Duong, Vireak Prom, Titya Thao, Lan Tran, Minh Phuc Vu, Lan Ngoc Vuong, David B Duong, Barbara Gottlieb

**Affiliations:** 1 Faculty of Medicine, University of Puthisastra, Phnom Penh, Cambodia; 2 Faculty of Medicine, University of Medicine and Pharmacy at Ho Chi Minh City, Ho Chi Minh City, Vietnam; 3 Institute of Health Sciences Education, McGill University, Montreal, Canada; 4 Hong Bang International University, Ho Chi Minh City, Vietnam; 5 Health Advancement in Vietnam, Beth Israel Deaconess Medical Center, Boston, Vietnam; 6 Division of Global Health Equity, Brigham and Women’s Hospital, Boston, USA; 7 Center for Primary Care, Harvard Medical School, Boston, USA; 8 Faculty of Medicine, Harvard Medical School, Boston, USA; 9 Social and Behavioral Sciences, Harvard TH Chan School of Public Health, Boston, USA; 10 Department of Medicine, Mass General Brigham Hospital, Boston, USA

**Keywords:** faculty development, virtual training, Cambodia, resource‑limited setting, LMICs, medical education, healthcare education reform

## Abstract

*Background:* Cambodia faces significant shortages of qualified physicians and skilled faculty, posing challenges for both medical training and educational reform. Innovative faculty development initiatives are essential in resource‑limited settings to address these gaps and support broader reforms.

*Objectives:* We describe a novel virtual faculty development program, created through regional and global collaboration, to enhance medical education in Cambodia.

*Methods:* We conducted a faculty development program consisting of six virtual workshops between August and September 2023. The program targeted basic science faculty in the pre‑clinical curriculum, introducing integrated teaching, case‑based learning methods, and related assessment strategies. Regional and international collaboration formed the backbone of the initiative, involving expertise from neighboring Vietnam and the United States.

*Findings:* Although participants were voluntary, and we offered no financial incentives, over 80% of the invited participants attended the sessions. Participants reported high satisfaction with the workshop content and format. Additionally, participants applied the concepts learned, as demonstrated by their creation and use of integrated clinical cases in their teaching.

*Conclusions:* Medical education is increasingly becoming a focus of global health collaboration. Findings highlight the feasibility and benefits of a partnership‑driven virtual faculty development model. By leveraging regional and international expertise to address local challenges, this initiative contributed to strengthening faculty capacity in resource‑constrained settings.

## Background

### Historical and current context of Cambodia

Cambodia, situated in Southeast Asia with a population of more than 17 million, became a lower‑middle‑income country in 2015. It inaugurated its first medical school in 1946 and achieved autonomy from French colonial rule in 1953. Unfortunately, this only medical school was closed from 1975 to 1979 during the Khmer Rouge genocide. Upon its reopening in 1980, fewer than 45 physicians had survived, with 20 of them having sought refuge overseas. This stark physician shortage, a dire consequence of the Khmer Rouge regime, has led to long‑term challenges for Cambodia’s healthcare and medical education systems, including the training of the future health workforce [1–3].

Currently, Cambodia has five medical schools, which collectively graduate approximately 600–700 physicians annually. Each institution enrolls between 100 and 350 new students per year, though this number varies by institution and available resources [4]. Despite these figures, the physician density in Cambodia remains critically low at 1.1 per 10,000 population, well below the Southeast Asia regional average of 7.7 per 10,000 people [5]. This shortage affects the accessibility and quality of healthcare services, leading to longer wait times, decreased patient satisfaction, and compromised health outcomes [6]. Beyond its immediate impact on healthcare, the shortage of physicians also reverberates throughout the medical education sector. With fewer physicians available to serve as full‑time educators and mentors, medical schools face challenges in providing comprehensive and high‑quality training to aspiring healthcare professionals. Cambodia’s five medical schools rely heavily on part‑time faculty, whose clinical responsibilities inevitably compete with their responsibilities as teachers. Moreover, Faculty lack protected time to improve their teaching skills, resulting in reliance on teaching and assessment methods known to be less effective for learning, such as large group lectures, rote memorization and fact‑based multiple‑choice questions.

### Medical education reform and faculty development

In response to the physician shortage and evolving healthcare needs, the Ministry of Health (MoH) of Cambodia initiated a national strategy in 2020 to transition all medical education programs from traditional content‑based models to Competency‑Based Medical Education (CBME). In 2022, the MoH revised and developed a new Cambodian Core Competency Framework (CCF) as the foundation for CBME reform. Following this, a comprehensive gap analysis was conducted, leading to the proposal of a new curriculum framework and the development of updated course syllabi. The new CBME‑based curriculum is planned for implementation in 2026, alongside its evaluation. Currently, efforts are focused on finalizing course syllabi, with ongoing support from local academic leaders and technical partners [4, 7].

A crucial component of this transformation is faculty development, which is a prerequisite for any successful curricular reform [8–11]. Initiatives tailored to local needs can directly support these efforts, ensuring that reforms are relevant, effective, and feasible. Faculty development encompasses a range of activities designed to improve the knowledge, skills, and professional abilities of academic staff. This includes formal training programs, workshops, mentorship, and opportunities for scholarly and clinical advancement [8]. The World Health Organization (WHO) and other international health bodies advocate for robust faculty development to enhance the quality of medical education globally. These organizations offer guidelines and frameworks to support the professional development of medical educators. Additionally, they emphasize the critical role of well‑trained faculty in achieving the health‑related sustainable development goals, as they are instrumental in producing competent health professionals [12].

Ironically, low‑ and middle‑income countries (LMICs) have both the greatest need and the greatest challenges in achieving high‑quality faculty development. Investing in faculty development can yield significant improvements in health profession education and healthcare delivery in these regions [13]. Enhanced faculty skills in LMICs, like Cambodia, which faces challenges such as limited resources, a shortage of trained professionals, and rapidly growing educational demands, may bridge the gap between current educational practices and desired international standards. Ultimately, this will foster a more competent health workforce capable of effectively addressing local health needs.

Although the importance of faculty development for medical educators in LMICs is well recognized, it is critical to develop approaches that recognize the many structural barriers. Strategies for faculty development in these countries must be appropriate, feasible, and sustainable. Although a small body of evidence supports the role of faculty development in enhancing the skills of faculty in LMICs, best practices for faculty development in global CBME implementation remain limited [13, 14]. We present a case study from Cambodia, where a national need to transition to a CBME curriculum was identified. In response, a virtual faculty development program was introduced, focusing on integrated teaching in CBME. This case study offers insights that are meaningful not only in Cambodia but also in other resource‑constrained settings. This article reports on a mixed‑methods evaluation of a virtual faculty development initiative, including survey‑based assessments of participant satisfaction, engagement, and application of training content.

## Methods

### Study setting

The University of Puthisastra (UP), located in Phnom Penh, was established in 2007. Its Faculty of Medicine, established in 2011, is one of five medical faculties in Cambodia. UP is dedicated to training healthcare professionals, including physicians, dentists, pharmacists, nurses, midwives, and laboratory technicians, among other fields. Despite challenges in funding, faculty shortages, and limited autonomy in curriculum planning given centralized control over curriculum content by the MOH, UP remains committed to academic excellence and innovative medical education.

This case study highlights one of UP’s initiatives: a virtual faculty development program that introduced case‑based learning (CBL) to basic science courses. The output was to train faculty in the introduction, design, and implementation of CBL sessions. The outcome was to enhance the teaching experience, increase student engagement, and prepare faculty for the upcoming CBME curriculum in line with national health education reforms.

### Learning collaborative in medical education: Local, regional and international actors

In 2022, with funding from the China Medical Board, a US‑based foundation, a medical education learning collaborative was formed among three medical schools along the Mekong River—UP (Cambodia), Can Tho University of Medicine and Pharmacy (Vietnam), and the University of Health Sciences Vientiane (Lao PDR). This collaboration was jointly led by the University of Medicine and Pharmacy at Ho Chi Minh City (UMP) and the Partnership for Health Advancement in Vietnam (HAIVN)—a partnership between Harvard Medical School (HMS) and its teaching hospitals, Brigham and Women’s Hospital and Beth Israel Deaconess Medical Center. Focused on medical education reform and CBME in the region, it aims to explore best practices, key CBME principles, and foster a collaborative environment for knowledge‑sharing and consensus‑building. During the first year (2022–2023), the collaborative, by consensus, focused on integrated teaching by implementing the CBL method within the pre‑clinical curriculum.

Integrated teaching is a fundamental component of CBME as it fosters the integration of basic and clinical sciences to enable learners to apply knowledge in real‑world contexts and develop essential competencies effectively [14, 15]. By breaking down disciplinary barriers, it promotes a holistic understanding of patient care, aligning with CBME’s goal of preparing learners for the complexities of modern healthcare. Integrating basic sciences with clinical medicine through organ‑based courses has been shown to enhance learner engagement, satisfaction, and critical thinking skills [16]. However, participants from UP identified several challenges, particularly the limited number of full‑time faculty, which hindered further implementation of this approach. They faced the challenge of conducting faculty development for part‑time faculty who were not technically employed by or under the supervisory control of UP.

### Faculty development program: design and implementation

In response to this challenge, the collaborative proposed to initiate a voluntary virtual faculty development program at UP. This program, consisting of six sessions, provided faculty with essential knowledge and skills for integrated teaching‑learning and CBL methodologies. The virtual format offered flexibility, allowing part‑time faculty to participate in their preferred time, but the program had no financial incentives. The sessions, held via Zoom, lasted 60 to 90 minutes and utilized a combination of didactic presentations and interactive discussions. Instructors were drawn from the faculty of the UMP in Vietnam and HMS in the United States.

The faculty development program was conducted from August 1 to September 5, 2023. It prioritized essential competencies that would enable faculty to adapt their teaching practices to align with the CBME approach. The sessions covered six topics as detailed in Table 1.

**Table 1:** Session titles, content overviews, and learning objectives.

SESSION	NAME OF THE TRAINING SESSION	TOPICS/CONTENTS INCLUDED IN THE TRAINING SESSION	LEARNING OBJECTIVES OF THE TRAINING SESSION
**1**	**Introduction to Competency‑Based Medical Education (CBME) and integrated teaching**	1. **What is CBME**, and how does this educational framework/ approach help us achieve our goals? 2. **What is integration**, and how does this educational approach help us achieve our goals? (vertical, horizontal, spiral integration; pre‑clinical and clinical integration)	Understand the overview of CBME and how it can be applied to their context Get an overview of integrated teaching in medical education and how it can be applied to their context
**2**	**CBME, Integrated module/course**	3. **The integrated module/course** The importance of integration in CBME Definition Integrated module/course Characteristics of the integrated module/course The structure of an integrated module/course Teaching & learning in the integrated module/course Assessment in the integrated module/course	Understand the importance, structure and operation of an integrated module/course
**3**	**Introduction to Case‑Based Learning (CBL)**	4. **CBL Introduction** The importance of CBL in CBME Building a case study for pre‑clinical & clinical phases Teaching by case study	Understand the importance of CBL in CBME Build a case study Build a teaching plan of CBL
**4**	**Clinical case design for CBL teaching**	5. **Stepwise approach for developing a case that integrates basic science and clinical knowledge.** Step by step in writing a case study, and example Briefly consider learning theory that supports integrated case‑based and problem‑based teaching and learning Tips for creating a qualified/effective case for CBL teaching and learning How to evaluate the qualified case for CBL teaching and learning	Analyze the steps to develop a clinical case for CBL teaching‑learning Identify the qualified case for CBL teaching and learning
**5**	**How to run a CBL session**	6. **How to teach a case study** Pre‑class In Class	Write a learning guide for students (including learning materials) Explain the principles of pre‑test Build a teaching plan Understand the skills of facilitating a case study in teaching
**6**	**Assessment in integration learning and teaching**	7. **How to assess/evaluate in an integrated module/course** Strategy of assessment Formative & summative assessment Assessment methods & tools Tasks of assessors	Build the assessment framework of an integrated module/course Choose the right assessment methods and tools Understand the assessors’ tasks

This faculty development program used task‑based performance assessment where each participant developed an integrated lesson relevant to their teaching course using the CBL method. A post‑training survey (available in the Supplementary Materials) was developed to evaluate the program. Guided by Kirkpatrick’s evaluation framework, the survey was refined through an iterative feedback process with the Monitoring and Evaluating team, who reviewed it for clarity, relevance, and alignment with the program’s objectives. The final survey included 17 Likert‑scale items assessing participants’ perceptions of the training’s organization, delivery, and overall effectiveness [8, 17]. It also evaluated the relevance of the content, time value, effectiveness of shared experiences, workshop pace, engagement opportunities, and the quality of facilitation, including feedback on presenters’ expertise and expert support.

Descriptive statistics were used to analyze the survey responses, with results presented as frequencies and percentages to summarize participant perceptions.

## Results

All 14 part‑time faculty members teaching basic science courses at UP were invited to the training workshop. Of these, 13 participated voluntarily, representing various courses such as anatomy, biochemistry, immunology, symptomatology, parasitology, physiology, and microbiology. Additionally, three faculty members with leadership roles at UP attended the program, bringing the total number of participants to 16. The training had a high participation rate throughout, with over 80% of the program participants attending each session.

**Figure 1:**
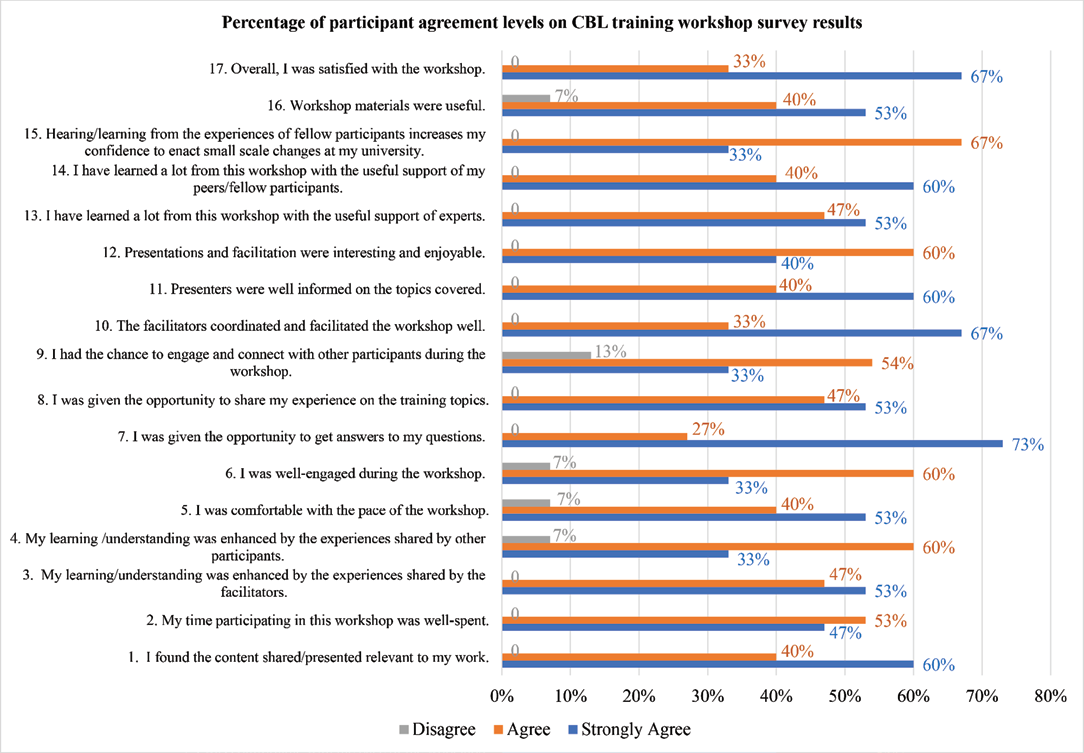
Percentage of participant agreement levels on CBL training workshop survey results.

Of the 16 participants, 15 responded to the post‑training survey. The survey results (see Table 2 and Figure 1) indicate a high level of satisfaction. Most respondents found the workshop content relevant to their professional responsibilities and considered their time investment well spent. The shared insights of facilitators and peers particularly enriched the learning experience for participants, with many expressing comfort with the pace of the workshop and the opportunities for engagement. In addition, participants appreciated the quality of coordination and facilitation, noting the expertise of the facilitators and the usefulness of the workshop materials. Overall, the workshop received positive feedback, with the majority expressing satisfaction and confidence in implementing the methods learned in their teaching practice.

**Table 2:** Summary of CBL training workshop survey results.

	RESPONSES (N = 15)					
	STRONGLY AGREE		AGREE		DISAGREE	
STATEMENTS	N	%	N	%	N	%
1. I found the content shared/presented relevant to my work.	9	60	6	40	0	0
2. My time participating in this workshop was well‑spent.	7	47	8	53	0	0
3. My learning/understanding was enhanced by the experiences shared by the facilitators.	8	53	7	47	0	0
4. My learning /understanding was enhanced by the experiences shared by other participants.	5	33	9	60	1	7
5. I was comfortable with the pace of the workshop.	8	53	6	40	1	7
6. I was well‑engaged during the workshop.	5	33	9	60	1	7
7. I was given the opportunity to get answers to my questions.	11	73	4	27	0	0
8. I was given the opportunity to share my experience on the training topics.	8	53	7	47	0	0
9. I had the chance to engage and connect with other participants during the workshop.	5	33	8	54	2	13
10. The facilitators coordinated and facilitated the workshop well.	10	67	5	33	0	0
11. Presenters were well informed on the topics covered.	9	60	6	40	0	0
12. Presentations and facilitation were interesting and enjoyable.	6	40	9	60	0	0
13. I have learned a lot from this workshop with the useful support of experts.	8	53	7	47	0	0
14. I have learned a lot from this workshop with the useful support of my peers/fellow participants.	9	60	6	40	0	0
15. Hearing/learning from the experiences of fellow participants increases my confidence to enact small‑ scale changes at my university.	5	33	10	67	0	0
16. Workshop materials were useful.	8	53	6	40	1	7
17. Overall, I was satisfied with the workshop.	10	67	5	33	0	0

Following the training, 12 out of the 13 part‑time faculty implemented the integrated case‑based teaching in their courses. We include a case example (Table 3) to illustrate how the faculty participants applied the lessons learned into practice in the context of Cambodia.

**Table 3:** Example case design for integrated learning by Dr. Youhok Lim, M.D, Anatomy lecturer at UP.

BROAD LEARNING OBJECTIVES	KEY CLINICAL CONCEPTS	KEY BASIC SCIENCE CONCEPTS	THE CASE	SAMPLE QUESTIONS/DISCUSSION
Diagnosis	Clinical examination of rotator cuff impingement (RCI) Imaging studies Diagnosis: Supraspinatus tendinopathy (ST), and differential diagnoses	Musculoskeletal anatomy of the shoulder: Rotator cuff muscles and their functions. Imaging mechanisms: How MRI, X‑rays, and ultrasound generate images of anatomical structures and tissues	A 52‑year‑old male worker presented with right shoulder pain, swelling, and limited movement, which began after heavy lifting two weeks ago On physical examination, there was resistance to abduction and tenderness when the clinician stood behind the patient and stabilized the scapula while the patient elevated the arm into full flexion with slight internal rotation and forearm pronation (video) An imaging study was obtained	What anatomical structures are likely involved in this patient’s shoulder pain? How can we differentiate this condition from bone fracture, osteoarthritis, joint capsulitis, and nerve injury? How does the physical examination help differentiate between different types of shoulder injuries? What is the cause of the limited range of motion? What imaging modalities would you use to confirm the diagnosis, and what findings would you expect?
Clinical course	Risk factors and progression of ST	Mechanism of mechanical injury: How mechanical forces cause injury. Histopathology of Impingement Lesions: The three stages of impingement lesions as proposed by Neer in 1983	Over the past two weeks, the patient’s symptoms have persisted despite rest and over‑the‑counter acetaminophen	What is the pathogenesis of impingement lesions?
Treatment	Treatment guidelines of ST	Painkiller mechanisms: How non‑steroidal anti‑inflammatory drugs (NSAIDs) and steroid injections alleviate pain. Muscle motion in physical therapy and exercises	The clinician gave some medications, but they were not fully effective	How do painkiller drugs work? Which alternative non‑operative procedures are indicated if oral medications are ineffective? And how do they work?
Prevention	Prevention strategies of shoulder injuries at work and in overhead athletes	Apply the concept of range of motion (ROM) to prevent injury	The patient inquired about preventing recurrence or other injuries	Please provide examples of how to maintain shoulder health and avoid movements that could cause pain or injury

In summary, participants reported high satisfaction with the training format and content, demonstrated engagement with learning activities, and applied core concepts through the creation and implementation of integrated teaching cases.

## Discussion

In response to the MOH’s strategy to shift from content‑based to CBME by 2026, significant changes in teaching and assessment methods are required. Our training program at UP is designed to support these national priorities by building foundational teaching competencies, thereby contributing to the ongoing transformation of medical education in Cambodia [7]. This initiative represents a significant step in empowering educators to lead transformative changes in medical education despite limited resources and other constraints.

The ongoing shortage of trained physicians impacts the quality of education in Cambodia, limiting the availability of full‑time educators and hindering continuous professional development. Our initiative sought to mitigate these challenges by offering flexible, virtual training; this approach was crucial in overcoming workforce constraints and ensuring that faculty could engage in professional development despite limited resources [13, 18]. Given Cambodia’s unique challenges, including a limited number of faculty spread across the country, a virtual platform was adopted. This approach aligns with current recommendations to leverage technology to build medical education capacity globally [9].

Faculty development is critical to improving the education of future physicians by providing faculty with the necessary tools, skills, and knowledge. In the short run, small‑scale, focused programs prioritizing areas of knowledge perceived as important by faculty, and designed for optimal convenience and aligned with national reform priorities can help advance faculty development. In our case, the training content was shaped directly by the Ministry of Health’s CBME reform directive, which identified teaching and assessment capacity as critical areas for development. While the program did not stem from a separate institutional needs assessment, it was responsive to an urgent, nationally recognized need. However, in the long run, medical schools, governments, international health organizations, and non‑governmental organizations must invest in faculty development, including financial resources to develop programs, identify and scale‑up best practices, and to provide faculty with protected time and other incentives to enhance their skills and knowledge. Our program highlights the value of targeted, small‑scale, faculty development initiatives that are responsive to broader system‑level reforms.

By focusing on integrated teaching and CBL, we addressed specific educational challenges and created immediate opportunities for faculty to apply newly acquired skills. The consistently high rate of participation underscores the importance of needs‑based faculty development tailored to the specific challenges and circumstances of medical schools in LMICs. Such programs can bridge the gap between existing educational practices and desired standards, thereby supporting the successful implementation of CBME [8, 19]. In addition, 12 out of the 13 basic sciences educator participants reported that the theoretical concepts learned were effectively applied, as evidenced by the CBL lessons created. This aligns with a Level 3 outcome—behavior change—under the Kirkpatrick model, which evaluates the application of learned skills and knowledge in practice [17]. The success of this program illustrates the feasibility of strengthening faculty capacity as a necessary component of addressing healthcare workforce demands, and contributing to improved educational quality and enhanced healthcare in Cambodia [9, 10, 20].

Similar faculty development efforts in other LMICs provide valuable insights that align with our findings. In Haiti, a targeted faculty development program in a rural teaching hospital demonstrated how small‑scale, context‑responsive initiatives can strengthen local teaching capacity [21]. In the Eastern Mediterranean region and sub‑Saharan Africa, evaluations of e‑learning programs confirmed that virtual or hybrid formats are effective in overcoming faculty shortages and infrastructure limitations when adapted to local settings [10, 22]. In Indonesia, a nationally developed faculty development model emphasized aligning training content with local educational gaps and institutional capacity, further supporting the need for contextually relevant interventions [19]. These cross‑country examples reinforce the relevance and transferability of our approach, particularly in leveraging virtual platforms, engaging part‑time faculty, and fostering sustainability in resource‑limited academic environments.

Collaboration was a key factor in the success of our program in Cambodia, which could be extrapolated to other settings in LIMCs. By partnering with experienced educators from the US (HMS) and Vietnam (UMP), we were able to overcome some of the constraints and enrich the training content. The involvement of these international experts not only provided valuable insights but also fostered a collaborative learning environment that benefited all participants. This model of collaboration demonstrates how partnerships between local, regional, and international institutions can enhance faculty development efforts in resource‑limited settings. By leveraging partnerships and adapting training content to local needs, this initiative offers a replicable approach for faculty development in other LMICs to achieve comparable outcomes [22, 23]. This innovative hybrid model of regional and global collaboration serves as a valuable example for LMICs in contextualizing content, scaling capacity‑building, and harmonizing international standards with local needs.

The program in this case study has several limitations. It involved a small number of faculty and addressed a specific area of medical education reform—the integration of clinical cases into basic science courses. Our evaluation focused primarily on process measures such as participation and participant reaction. While the high rates of case development and implementation by participants are encouraging, future research should examine higher level outcomes, including the long‑term impact on faculty teaching practices and student learning outcomes—Level 4 of the Kirkpatrick model [17]. Despite these limitations, the consistent rates of participation and satisfaction, along with the successful application of integration techniques, particularly in the absence of external incentives, suggest that this approach is both feasible and potentially highly acceptable in a resource‑limited context.

## Conclusion

Medical education is increasingly a focus of global health collaboration. This virtual faculty development program demonstrates the feasibility and impact of an innovative collaboration model among local, regional, and global institutions. It provides a practical approach to addressing educational and healthcare workforce challenges in resource‑limited settings. By equipping faculty with essential teaching skills, the program lays the foundation for broader educational reforms, supports advancements toward competency‑based frameworks, and contributes to the transformation of medical education in Cambodia, offering a replicable model for similar LMIC contexts. Future directions include expanding the program to additional medical schools in Cambodia and exploring further faculty development initiatives to support the transition to CBME.

## Data Availability

De‑identified data and workshop materials generated during this study are available from the corresponding author upon reasonable request.

## Additional File

The additional file for this article can be found as follows:

Supplementary material. Post‑Training Survey Form.

## References

[r1] McGrew L. Health Care in Cambodia. Cultural Survival. March 2, 2010. Accessed May 28, 2024. https://www.culturalsurvival.org/publications/cultural-survival-quarterly/health-care-cambodia.

[r2] Santini H. Rebirth of the health‑care system in Cambodia. Lancet. 2002;360(Special Issue):s57–s58. doi:10.1016/s0140-6736(02)11824-1. 12504507

[r3] The World Bank. Population, Total ‑ Cambodia. 2022. Accessed May 28, 2024. https://data.worldbank.org/indicator/SP.POP.TOTL?locations=KH.

[r4] Lim S, Cheab S, Goldman LN, Ith P, Bounchan Y. The past, present and future of medical education in Cambodia. Med Teach. 2024;46(6):842–848. doi:10.1080/0142159X.2024.2327490. 38493077

[r5] WHO. World Health Statistics 2023. Monitoring Health for the SDGs Sustainable Development Goals HEALTH FOR ALL; 2023. https://www.who.int/publications/book-orders.

[r6] Annear PL, Jacobs B, Nachtnebel M. The Kingdom of Cambodia Health System Review. Manila: World Health Organization, Regional Office for the Western Pacific; 2015.

[r7] World Bank. Strengthening Pre‑Service Education System for Health Professionals Project. World Bank. May 29, 2020. Accessed May 23, 2024. https://projects.worldbank.org/en/projects-operations/project-detail/P169629.

[r8] Steinert Y, Mann K, Centeno A, et al. A systematic review of faculty development initiatives designed to improve teaching effectiveness in medical education: BEME Guide No. 8. Med Teach. 2006;28(6):497–526. doi:10.1080/01421590600902976. 17074699

[r9] Frenk J, Chen L, Bhutta ZA, et al. Health professionals for a new century: Transforming education to strengthen health systems in an interdependent world. Lancet. 2010;376(9756):1923–1958. doi:10.1016/S0140-6736(10)61854-5. 21112623

[r10] Kumar A, Atwa H, Shehata M, Al Ansari A, Deifalla A. Faculty development programmes in medical education in the Eastern Mediterranean Region: A systematic review. East Mediterr Health J. 2022;28(5):362–380. doi:10.26719/emhj.22.014. 35670441

[r11] Tran TD, Vu PM, Pham HTM, et al. Transforming medical education to strengthen the health professional training in Viet Nam: A case study. Lancet Reg Health West Pac. 2022;27:100543. doi:10.1016/j.lanwpc.2022.100543. 35874914 PMC9301568

[r12] WHO. Global Strategy on Human Resources for Health: Workforce 2030. WHO; 2020. 10.1186/s12960-024-00940-x PMC11450981 39363378

[r13] Keiller L, Nyoni C, van Wyk C. Online faculty development in low‑ and middle‑income countries for health professions educators: A rapid realist review. Hum Resour Health. 2022;20(1):12. doi:10.1186/s12960-022-00711-6. 35093081 PMC8799968

[r14] Frank JR, Snell LS, Cate OT, et al. Competency‑based medical education: Theory to practice. Med Teach. 2010;32(8):638–645. doi:10.3109/0142159X.2010.501190. 20662574

[r15] Harden RM. The integration ladder: A tool for curriculum planning and evaluation. Med Educ. 2000;34(7):551–557. doi:10.1046/j.1365-2923.2000.00697.x. 10886638

[r16] McLean SF. Case‑based learning and its application in medical and health‑care fields: A review of worldwide literature. J Med Educ Curric Dev. 2016:3:JMECD.S20377. doi:10.4137/JMECD.S20377. PMC5736264 29349306

[r17] Kirkpatrick DL, Kirkpatrick JD. Evaluating Training Programs: The Four Levels. 3rd ed. Berrett‑Koehler Publishers; 2006.

[r18] Hu WCY, Nguyen VAT, Nguyen NT, Stalmeijer RE. Becoming agents of change: Contextual influences on medical educator professionalization and practice in a LMIC context. Teach Learn Med. 2023;35(3):323–334. doi:10.1080/10401334.2022.2056743. 35465797

[r19] Ambarsarie R, Mustika R, Soemantri D. Formulating a need‑based faculty development model for medical schools in Indonesia. Malays J Med Sci. 2019;26(6):90–100. doi:10.21315/mjms2019.26.6.9. PMC6939731 31908590

[r20] Tumlinson K, Jaff D, Stilwell B, Onyango DO, Leonard KL. Reforming medical education admission and training in low‑ and middle‑income countries: Who gets admitted and why it matters. Hum Resour Health. 2019;17(1):91. doi:10.1186/s12960-019-0426-9. 31791358 PMC6889551

[r21] Hudspeth JC, Gangasani N, Julmisse M, et al. Piloting a faculty development program in a rural Haitian teaching hospital. Ann Glob Health. 2022;88(1):19. doi:10.5334/aogh.3512. 35433286 PMC8916063

[r22] Talaat W, Dalen V, Hamam A, Khamis N, Abdel Nasser A. Evaluation of the joint master of health professions education: A distance learning program between Suez Canal University, Egypt, and Maastricht University, The Netherlands. Intel Prop Rights. 2014;2(1):107. doi:10.4172/2375-4516.1000107.

[r23] Mennin S, Kalishman S, Eklund MA, Friedman S, Morahan PS, Burdick W. Project‑based faculty development by international health professions educators: Practical strategies. Med Teach. 2013;35(2):e971–e977. doi:10.3109/0142159X.2012.731096. 23102155

