# Supplementary material for: Strengthening Faculty Development through Regional and Global Collaboration: An Innovative Virtual Program in Cambodia
Source: Ann Glob Health. 2025 Jun 10;91(1):29. doi: 10.5334/aogh.4660 (PMC12164755; doi:10.5334/aogh.4660)
Supplement: Supplementary material. — Post‑Training Survey Form. [file agh-91-1-4660-s1.pdf]

Post-Training Survey Form

Content	Strongly Disagree	Disagree	Agree	Strongly Agree
1. I found the content shared/presented relevant to my work				
2. My time participating in this workshop was well-spent				
3. My learning/understanding was enhanced by the experiences shared by the facilitators				
4. My learning /understanding was enhanced by the experiences shared by other participants				
5 I was comfortable with the pace of the workshop				
6. I was well-engaged during the workshop				
7. I was given the opportunity to get answers to my questions				
8. I was given the opportunity to share my experience on the training topics				
9. I had the chance to engage and connect with other participants during the workshop				
10. The facilitators coordinated and facilitated the workshop well				
11. Presenters were well informed on the topics covered				
12. Presentations and facilitation were interesting and enjoyable				
13. I have learned a lot from this workshop with the useful support of experts				
14. I have learned a lot from this workshop with the useful support of my peers/fellow participants				
15. Hearing/learning from the experiences of fellow participants increases my confidence to enact small scale changes at my university				
16. Workshop materials were useful				
17. Overall, I was satisfied with the workshop				
